# Astaxanthin in Cardiovascular Health and Disease

**DOI:** 10.3390/molecules17022030

**Published:** 2012-02-20

**Authors:** Robert G. Fassett, Jeff S. Coombes

**Affiliations:** 1 School of Medicine, The University of Queensland, Brisbane, Queensland, 4000, Australia; 2 Department of Renal Medicine, Royal Brisbane and Women’s Hospital, Brisbane, Queensland, 4029, Australia; 3 The School of Human Movement Studies, The University of Queensland, St Lucia, Queensland, 4072, Australia; Email: jcoombes@uq.edu.au

**Keywords:** antioxidants, marine carotenoids, inflammation, oxidative stress, cardiovascular disease

## Abstract

Oxidative stress and inflammation are established processes contributing to cardiovascular disease caused by atherosclerosis. However, antioxidant therapies tested in cardiovascular disease such as vitamin E, C and β-carotene have proved unsuccessful at reducing cardiovascular events and mortality. Although these outcomes may reflect limitations in trial design, new, more potent antioxidant therapies are being pursued. Astaxanthin, a carotenoid found in microalgae, fungi, complex plants, seafood, flamingos and quail is one such agent. It has antioxidant and anti-inflammatory effects. Limited, short duration and small sample size studies have assessed the effects of astaxanthin on oxidative stress and inflammation biomarkers and have investigated bioavailability and safety. So far no significant adverse events have been observed and biomarkers of oxidative stress and inflammation are attenuated with astaxanthin supplementation. Experimental investigations in a range of species using a cardiac ischaemia-reperfusion model demonstrated cardiac muscle preservation when astaxanthin is administered either orally or intravenously prior to the induction of ischaemia. Human clinical cardiovascular studies using astaxanthin therapy have not yet been reported. On the basis of the promising results of experimental cardiovascular studies and the physicochemical and antioxidant properties and safety profile of astaxanthin, clinical trials should be undertaken.

## 1. Introduction

Oxidative stress and inflammation are established pathophysiological processes involved with the development of atherosclerotic cardiovascular disease. Consequently antioxidant therapies such as vitamin E, C and β-carotene have been assessed in clinical trials in patients at risk of cardiovascular events. With the exception of the hemodialysis population where the Secondary Prevention with Antioxidants of Cardiovascular disease in End stage renal disease (SPACE) study showed a reduction in cardiovascular events and mortality with the use of vitamin E, the majority of other studies have been unsuccessful. Hence, new, more potent and effective antioxidant therapies have been sought. One such agent is astaxanthin, which has antioxidant and anti-inflammatory effects. It is a more potent quencher of singlet oxygen [1] than other antioxidants and its polar properties allows strategic placement in cell membranes [2]. We will review the evidence this nutraceutical is worthy of further investigation in the prevention and treatment of atherosclerotic cardiovascular disease. This will expand and update our previous reviews and that of others in this area [3,4,5].

## 2. Oxidative Stress and Inflammation

Oxidative stress and inflammation are established cardiovascular risk factors supplementing traditional ones that include hyperlipidemia, family history, smoking and hypertension [6]. Antioxidants in the diet and as supplements decrease lipid and protein oxidation and attenuate the progression of atherosclerosis [7,8,9]. There is an association between the intake of antioxidants, their plasma levels, and cardiovascular event reduction, which supports the tenant that oxidative stress is involved as a mechanism in the development of vascular disease caused by atherosclerosis [10,11,12,13,14,15]. In addition, a reduction in the dietary intake of antioxidants is associated with the presence of oxidative stress and inflammation [16]. Studies assessing the dietary intake or supplementation with vitamin E and β-carotene have shown that higher intake is associated with less cardiovascular disease [11,17,18,19,20,21,22]. Potent dietary antioxidants such as astaxanthin have not been well investigated in this context.

Most studies where antioxidants have been used as an intervention have failed to show any cardiovascular or mortality benefit [23,24,25]. This may not be due to lack of efficacy of the antioxidant but could be because at risk participants have not been selected based on the presence of confirmed oxidative stress. Some studies where participants were highly likely but not proven to have oxidative stress have shown a benefit from antioxidant therapy [26,27,28]. Additional intervention trials assessing more potent antioxidants in populations with proven oxidative stress at entry should be conducted.

## 3. Carotenoids

Humans cannot synthesize carotenoids and must ingest them in the diet from sources such as algae, plants and fungi [29]. Based on their chemical structure, carotenoids are classified into two types: Carotenes and xanthopylls. Lycopene and β-carotene are examples of carotene carotenoids and lutein, canthaxanthin, zeaxanthin, violaxanthin, capsorubin and astaxanthin are xanthopyll carotenoids [2,30].

Carotenoids exert varying effects according to their polarity and hence, how they configure with cellular membranes [2]. Lycopene and β-carotene, which are non-polar and produce disorder of the membrane structure and oxidation of lipids in a polyunsaturated fatty acid enriched membrane model in contrast to the polar astaxanthin, which preserves the structure of the membrane [31]. These contrasting effects may explain the different outcomes seen in clinical and experimental studies. In some clinical trials the non-polar β-carotene had no beneficial effects on cardiovascular disease [32,33,34,35,36] and was actually pro-oxidant at higher doses [37]. In experimental studies the polar astaxanthin has myocardial preservation effects but this is yet to be confirmed in human clinical trials [38,39,40].

## 4. Astaxanthin

The keto-carotenoid astaxanthin has enhanced antioxidant properties and is a potent quencher of singlet oxygen [1]. This may relate to its structure [41] and account for its increased potency compared with β-carotene [42,43]. The chemistry and structural formulae of astaxanthin and other carotenoids has been reviewed elsewhere [30].

Astaxanthin is found in a variety of living organisms, many of which are found in the marine environment. Here it is found in varying concentrations in unicellular microalgae, plankton, krill and other seafood such as salmon, trout and crustaceans, including crayfish and prawns. It gives the latter group their reddish colour [44]. It is also present in some fungi such as *Phaffia rhodozyma or Xanthophyllomyces dendrorhous*, complex plants, flamingo feathers and the retina of quail [44]. The United States Food and Drug Administration approved the use of astaxanthin as a feed additive for aquaculture in 1987 and subsequently in 1999 astaxanthin was approved as a nutraceutical [41]. Humans cannot manufacture astaxanthin and ingested astaxanthin cannot be converted to vitamin A therefore excessive intake will not lead to hypervitaminosis A [45,46]. Astaxanthin is a potent quencher of free radicals and reactive oxygen and nitrogen species [1,42,47]. It is 11 times more potent as a singlet oxygen quencher than β-carotene and 550 times greater than alpha tocopherol [1,42,47]. Both its high potency and polar properties make astaxanthin an attractive nutraceutical for further investigation in atherosclerotic cardiovascular disease where antioxidant cellular protection may be of clinical benefit [48].

## 5. Sources of Astaxanthin

Astaxanthin is most commonly harvested from the unicellular microalgae, *Haematococcus pluvialis* [49]. This contains a mixture of configurational isomers. From this source astaxanthin is produced in large quantities compared with *Xanthophyllomyces dendrorhous*, formerly *Phaffia rhodozyma*, krill and shrimp [50]. These other sources of commercial astaxanthin production include *Euphausia pacifica* (Pacific krill), *Euphausia superba* (Antarctic krill), *Pandalus borealis* (shrimp) and *Xanthophyllomyces dendrorhous*. Astaxanthin composition differs between these organisms based on the stereoisomer content. Astaxanthin produced by *haematococcus pluvialis*, consists of the (3-*S*,3'-*S*) stereoisomer. This is most frequently used as a feed supplement in aquaculture and therefore is most commonly ingested by humans.

**Figure 1:**
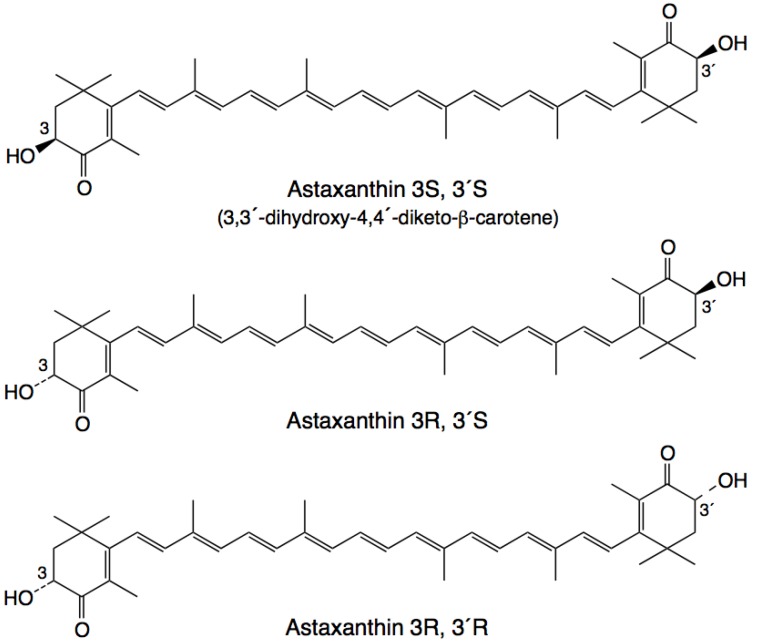
Stereoisomers of astaxanthin [41].

**Figure 2:**
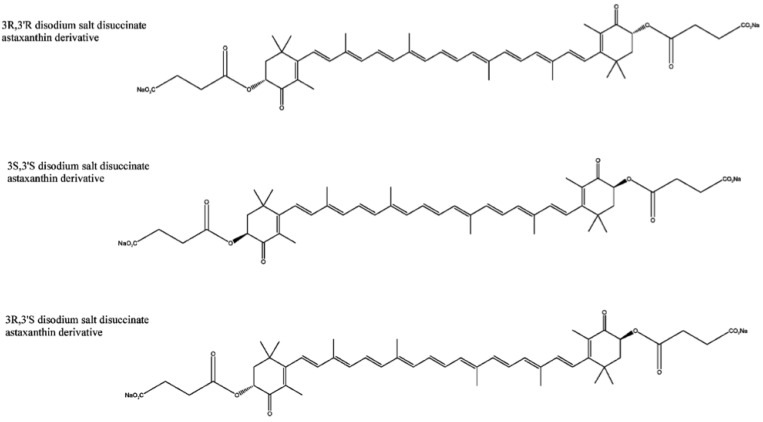
Three stereoisomers of the derivative DDA [40].

Astaxanthin has three stereoisomers (Figure 1): (3*R*,3'*R*), (3*R*,3'*S*) and (3*S*,3'*S*) [41]. A synthetic derivative of astaxanthin has been produced by Cardax Pharmaceuticals called disodium disuccinate astaxanthin (DDA), which consists of the three stereoisomers (Figure 2) [40]. DDA has been used predominantly in experimental myocardial ischaemia-reperfusion models [38]. DDA reportedly had greater aqueous solubility, which would allow for intravenous as well as oral administration. However, DDA is no longer available and Cardax Pharmaceuticals produces another astaxanthin derivative, the prodrug Heptax/XanCor, CDX-085 [51]. The exact nature of the prodrug has not been published but may represent compounds listed in a patent from Cardax Pharmaceuticals [52]. The main utility targeted for this pharmaceutical company pipeline drug is the prevention of re-thrombosis, reduction of triglycerides, amelioration of the metabolic syndrome, and treatment of liver inflammatory disease [53]. In addition, this compound is claimed to have improved water dispersibility and greater bioavailability than DDA and astaxanthin from* Haematococcus pluvialis*. CDX-085 is hydrolysed in the intestine releasing free astaxanthin for absorption. So far only one study using CDX-085 in animals has been reported [54].

Insufficient evidence is available to direct us regarding the relative therapeutic merits of astaxanthin or its derivatives such as DDA. However, an astaxanthin derivative such as DDA would be required for any potential intravenous indication. The therapeutic benefit of astaxanthin and its derivatives may result in different outcomes depending on the applied stereoisomer [55].

## 6. Experimental Studies Relevant to the Cardiovascular System Using Astaxanthin

Table 1 summarises the experimental studies, relevant to the cardiovascular system, that have used astaxanthin. In non-cardiovascular disease models, astaxanthin attenuates oxidative stress and inflammation, important pathophysiological mechanisms in atherosclerotic cardiovascular disease [56,57,58,59,60,61,62,63,64,65,66,67] In other studies, astaxanthin decreased lipid peroxidation [68], inflammation [59,60,65,66], thrombosis [54] and scavenger receptor expression and macrophage matrix metalloproteinase activity [69]. Astaxanthin also decreased plaque macrophage infiltration, improved plaque stability and decreased apoptosis in the atheroma of hyperlipidemic rabbits [70]. In addition, astaxanthin decreased the size of fat cells, improved adiponectin levels, increased HDL and decreased plasma triglycerides and non-esterified fatty acids in SHR/NDmcr-*cp* (*cp/cp*) rats [71]. Astaxanthin also reduced blood pressure and increased insulin sensitivity in the rat [72].

### Cardiovascular Studies

The myocardial ischemia-reperfusion model has been used in several animals including the rat, rabbit and dog to assess the efficacy of prior treatment with DDA in attenuating myocardial damage [39,40,73,74]. Myocardial infarct size was significantly decreased in Sprague-Dawley rats with four-days of prior treatment with intravenous DDA at doses of 25, 50 and 75 mg/Kg/d [39]. The effects were dose related, with less myocardial damage at higher doses. In another study conducted in rat investigators assessed the effects of pre-treatment with orally administered DDA, 125 and 500 mg/kg/d for seven days on metabolised astaxanthin concentrations from DDA in myocardial muscle tissue [40]. Astaxanthin concentrations were 400 nM after 125 mg/kg/d and 1,634 nM after 500 mg/kg/d.

**Table 1:** Animal investigations of astaxanthin effects related to the cardiovascular system.

Study	Model	Dose	Duration/timing of supplementation	Effects of (metabolized) astaxanthin
Lauver *et al.* 2008 [38]	Dog (occlusive carotid artery thrombus)	IV DDA 10, 30, or 50 mg/kg/body weight	30 mins after occlusion	- Reduced incidence of secondary thrombosis
Aoi *et al.* 2003 [61]	C57BL/6 mice	Diet supplemented with astaxanthin 0.02% weight/weight and food intake recorded	3 weeks	- Attenuation of exercise increased 4-hydroxy-2-nonenal-modified protein and 8-hydroxy-2′-deoxyguanosine in cardiac and gastrocnemius muscle
				- Attenuation of exercise increases in creatine kinase and myeloperoxidase activity in cardiac and gastrocnemius muscle
				- Astaxanthin accumulated in cardiac and gastrocnemius muscle
Gross and Lockwood 2004 [39]	Myocardial infarct model Sprague-Dawley rats	DDA 25/50/75 mg/kg intravenously daily	4 days prior to myocardial infarction	- Myocardial infarct size significantly reduced
Li *et al* 2004 [70]	WHHL rabbits	100 mg astaxanthin/kg feed	24 weeks	Reduced macrophage infiltration into plaque, improved plaque stability and decreased apoptosis
Hussein *et al.* 2005 [75]	Stroke prone Spontaneously hypertensive rats	50 mg/kg body weight/day	5 weeks	- Significant blood pressure reduction
				- Delayed incidence of stroke
Lauver *et al.* 2005 [76]	Rabbit model of myocardial ischemia/reperfusion	DDA 50 mg/kg/day intravenously	5 days	- Significant reduction in complement activation
				- Significant reduction in myocardial infarct size
Gross *et al.* 2005 [74]	Canine model of myocardial ischemia/reperfusion	DDA 50 mg/kg/day intravenously	2 h or daily for 4 days	- Significant reduction in myocardial infarct size
				- Two of three dogs treated for four days had 100% cardiac protection
Gross *et al.* 2006 [40]	Sprague-Dawley rats Left anterior descending coronary artery occlusion/reperfusion	DDA 125 or 500 mg/kg/day orally	7 days	- Astaxanthin loading of myocardium indicating good bioavailability
				- Trends in lowering of lipid peroxidation products
				- Significant reduction in myocardial infarct size
Hussein *et al.* 2006 [77]	Spontaneously hypertensive rats	5 mg/kg body weight/day	7 days	- Significant reduction in nitric oxide end products
				- Significant reduction in elastin bands in aorta
				- Significant reduction in wall/lumen arterial ratio in coronary arteries
Hussein *et al* 2006 [71]	SHR/NDmcr- *cp* rats	Astaxanthin 50 mg/kg/d	22 weeks	Astaxanthin significantly reduced BP, fasting BSL, insulin resistance and sensitivity, triglyceride and non-esterified fatty acid levels. Astaxanthin decreased fat cell size
Kishimoto *et al* 2009 [69]	Human monocytic cell line THP-1	Astaxanthin 5–10 μM	24 h	Astaxanthin inhibits activation of macrophages
Nakao *et al.* 2010 [78]	BALC/c mice	Astaxanthin 0, 0.02, 0.08% orally/day	8 weeks	- No change in blood glutathione concentration
				- No change in lymphocyte mitochondrial membrane potential
				- Higher myocardial mitochondrial membrane potential and contractility index
Khan *et al.* 2010 [54]	C57BL/6 mice	CDX-085 500 mg/kg/d	14 days	- Free astaxanthin present in the plasma, heart, liver and platelets
				- Significantly increased basal arterial blood flow and delay in occlusive thrombosis after endothelial injury
	Human umbilical vein endotheilial cells and platelets from Wistar-Kyoto rats			- Significantly increased release of nitric oxide and decreased peroxynitrite levels
Aduri *et al.* 2011 [79]	Rat	VitaePro 70 mg/kg BW (Containing astaxanthin 2%)	21 days	- Significantly reduced myocardial infarct size
				- Significantly reduced apoptosis and oxidative stress

VitaePro-astaxanthin zeaxanthin and luetin. WHHL-Watanabe heritable hyperlipidemic rabbits.

Lipid peroxidation products were reduced. Using a similar model in the rabbit prior treatment with intravenous DDA 50 mg/kg/d for four-days also significantly reduced myocardial infarct size and an improved the salvage of myocardium [76]. Treated animals also had reduced activation of complement and decreased inflammation [76]. Dogs treated with intravenous DDA for four-days or alternatively two hours prior to left anterior descending coronary artery occlusion both had significantly decreased myocardial infarct size [74]. In fact two out of four dogs treated for four days prior had full protection of their myocardium [74].

A separate group of investigators assessed combination therapy (VitaePro) which contains three carotenoids including astaxanthin (2%), zeaxanthin (1.23%) and lutein (8.1%) in safflower oil, in a myocardial ischaemia reperfusion model in the rat [79]. Prior orally administered VitaePro for 21 days resulted in significant cardioprotection compared with control and vitamin E treated groups.

As hypertension is a significant cardiovascular risk factor the effects of astaxanthin on blood pressure may be significant in the assessment of its effects on the cardiovascular system. Astaxanthin administered orally to spontaneously hypertensive rats (SHR) resulted in a significant blood pressure (BP) reduction over after 14-days. This effect was not seen in normotensive Wistar Kyoto rats [75]. Five-weeks of orally administered astaxanthin in stroke prone SHR also resulted in a significant reductions in BP [75]. Nitric oxide induced vascular relaxation was also enhanced in the aortas of the rats [75]. The mechanism of BP reduction may be through reduction in nitric oxide end products as demonstrated in experiments in SHR, where orally administered astaxanthin significantly decreased these [77]. Astaxanthin may also attenuate atherosclerosis by reducing wall/lumen ratio in coronary arteries and decreasing elastin bands in the aorta [77].

Orally administered CDX-085 given to C57BL/6 mice produced metabolised astaxanthin from CDX-85 in plasma, heart, liver and platelets and these mice had significantly enhanced arterial blood flow and delayed occlusive thrombosis as a consequence of endothelial injury [54]. Human umbilical vein endothelial cells and platelets isolated from Wistar-Kyoto rats treated with astaxanthin had significantly increased nitric oxide release and decreased peroxynitrite levels suggesting a role for astaxanthin in thrombosis prevention [54].

Astaxanthin attenuated exercise-induced oxidative stress in C57BL/6 mice [61]. Myeloperoxidase, 8-hydroxy-2′-deoxyguanosine and 4-hydroxy-2-nonenal-modified protein were reduced in cardiac and gastrocnemius muscle [61]. In addition, astaxanthin improved cycling time trial performance in a randomised controlled trial conducted in 21 cyclists [80].

Female BALB/c mice that received astaxanthin for eight-weeks had dose dependent increases in plasma astaxanthin had higher cardiac mitochondrial membrane potential and contractility index compared with control animals suggesting astaxanthin provides myocardial protection [78]. DDA administered to dogs using a carotid artery thrombosis model showed dose-dependent reductions in re-thrombosis and reduced re-thrombosis after thrombolysis [38].

## 7. Human Astaxanthin Studies

There have not been any clinical studies specifically addressing cardiovascular outcomes using astaxanthin. However, there are studies assessing astaxanthin in healthy subjects and other disease states. Some of these are summarised in Table 2. Most of the studies assessed astaxanthin bioavailability, dosing and safety and others assessed its effects on oxidative stress. The combination of lutein, zeaxanthin and astaxanthin was studied in age-related macular degeneration where there were improvements or stabilization of visual acuity, contrast sensitivity and visual function [81]. Studies have also been conducted in patients with reflux oesophagitis, and overweight and obesity, where measurements of biomarkers of oxidative stress and inflammation were included.

### 7.1. Bioavailability

The bioavailability of astaxanthin derived from different sources in the marine environment was investigated in 28 volunteers in a randomised controlled double blind trial [82]. Participants received either 250 g of wild or aquaculture salmon (5 µg/g) in their diet. In the wild, salmon ingest astaxanthin from krill whereas aquacultured salmon ingest it from feed additive containing astaxanthin. Plasma astaxanthin concentrations were greater after ingesting salmon sourced from aquaculture than salmon from the wild. Plasma concentrations of the astaxanthin (3-*S*,3′-*S*) isomer were higher than the proportionate concentration of this isomer in the salmon suggesting astaxanthin isomers may have differing bioavailability. After ingestion of single oral dose of 10 mg of astaxanthin and 100 mg over four-weeks there was a non-linear dose response and selective absorption of *Z*-isomers and the plasma elimination half-life was 52 (SD 40) h [83]. Erythrocyte astaxanthin and plasma carotenoids were significantly higher after twelve weeks of 3 mg/d of orally administered astaxanthin compared with 1 mg/d or placebo [84,85].

### 7.2. Dosing

Human clinical studies have used orally administered astaxanthin in a dose that ranges from 4 mg up to 100 mg/day, given from a one off dose up to durations of two-year (Table 2).

### 7.3. Safety

The safety of orally administered astaxanthin derived from *haematococcus pluvialis* was assessed in healthy volunteer adults in a randomised double blind placebo-controlled trial [86]. Participants received either astaxanthin 6mg/d or identical placebo for eight-weeks. There were no adverse events or significant BP and biochemical changes noted. In another study conducted in 20 healthy males 6 mg/d of orally administered astaxanthin for ten days decreased whole blood transit time [87]. High concentrations of astaxanthin added *in vitro* to blood taken from 20 volunteers, eight of who were taking aspirin, showed no adverse effects on platelet, coagulation and fibrinolytic function [88]. There have been no adverse events of any consequence, associated with the administration of astaxanthin, reported to date, in clinical trials in humans.

### 7.4. Oxidative Stress and Inflammation

Healthy human volunteers and patients with reflux oesophagitis treated with orally administered astaxanthin have significantly decreased oxidative stress and inflammation [68,89,90]. Twenty-four healthy volunteers took astaxanthin 1.8–21.6 mg/d for two weeks and LDL lag time which is a measure of LDL susceptibility to oxidation, was significantly greater in those taking astaxanthin [68]. 

**Table 2:** Clinical studies investigating the safety, bioavailability and effects of astaxanthin on oxidative stress.

Study	Study population (n = subject numbers)	Dosage of astaxanthin	Study design	Duration of supplementation	Effects of astaxanthin
Iwamoto *et al.* 2000 [68]	Volunteers (n = 24)	Different doses: 1.8, 3.6, 14.4, 21.6 mg/day	Open labelled	2 weeks	- Reduction of LDL oxidation
Osterlie *et al.* 2000 [91]	Middle aged male volunteers (n = 3)	100 mg	Open labelled	Single dose	- Astaxanthin taken up by VLDL chylomicrons
Mercke Odeberg *et al.* 2003 [92]	Healthy male volunteers (n = 32)	40 mg	Open labelled parallel	Single dose	- Enhanced bioavailability with lipid based formulation
Spiller *et al.* 2003 [86]	Healthy adults (n = 35)	6 mg/day (3 × 2 mg tablets/day)	Randomised, double blind, placebo controlled	8 weeks	- Demonstrated safety assessed by measures of blood pressure and biochemistry
Coral-Hinostroza *et al.* 2005 [83]	Healthy adult males (n = 3)	10 mg and 100 mg	Open labelled	Single dose or 4 weeks	- Cmax 0.28 mg/L at 11.5 h at high dose and 0.08 mg/L at low dose
					- Elimination half life 52+/− 40 hours
					- *Z* -isomer selectively absorbed
Karppi *et al.* 2007 [89]	Healthy non-smoking Finnish males (n = 40)	8 mg/day	Randomised, double blind, placebo controlled	12 weeks	- Intestinal absorption adequate with capsules
					- Reduced levels of plasma 12 and 15 hydroxy fatty acids
					- Decreased oxidation of fatty acids
#Parisi *et al.* 2008 [93]	Non-advanced age related macular degeneration (n = 27)	4 mg/day	Randomised controlled trial open labelled no placebo	12 months	- Improved central retinal dysfunction in age related macular degeneration when administered with other antioxidants
Miyawaki *et al.* 2008 [87]	Healthy males (n = 20)	6 mg/day	Single blind, placebo controlled	10 days	- Decreased whole blood transit time (improved blood rheology)
Rufer *et al.* 2008 [82]	Healthy males (n = 28)	5μg/g salmon flesh (wild *vs.* aquacultured)	Randomised, double blind, placebo controlled	4 weeks	- Bioavailability initially better with aquacultured salmon but equivalent at day 28
					- Isomer (3, *S,* 3′ *S* ) greater in plasma compared with isomer proportion in salmon flesh
Uchiyama *et al.* 2008 [94]	Healthy volunteers at risk of metabolic syndrome n = 17	8 mg twice daily	Uncontrolled open-labelled	3 months	- Significantly decreased HbA1c and TNF-alpha
					- Significantly increased adiponectin
Park *et al.* 2010 [95]	Healthy females (n = 14)	0, 2, 8 mg/day	Randomised, double blind, placebo controlled	8 weeks	- Decreased plasma 8-hydroxy-2′-deoxyguanosine after four weeks
					- Lower CRP after four weeks in those taking 2 mg/day
Yoshida *et al.* 2010 [96]	Hypertriglyceridemic males and females n = 61	0, 6, 12, 18 mg/day	Randomised double blind placebo controlled trial	12 weeks	- Significantly decreased triglycerides and increased HDL cholesterol
					- Significantly increased adiponectin
Choi *et al.* 2011 [97]	Overweight and obese males and females n = 23	5 mg or 20 mg/day	Randomised double blinded trial	3 weeks	- Significantly decreased oxidative stress biomarkers (MDA, ISOP, SOD and TAC)
*Piermarocchi S *et al* . 2011 [81]	Non-advanced age related macular degeneration (n = 145)	4 mg/day	Randomised controlled trial open labeled, no placebo	2 years	Stabilized or improved visual acuity, contrast sensitivity and visual function

* This is an extension of the Parisi V study [93] #.

In 40 healthy non-smoking Finnish males taking astaxanthin plasma 12- and 15-hydroxy fatty acid levels were significantly reduced which suggests astaxanthin decreases fatty acid oxidation [89]. Fourteen healthy females received dietary astaxanthin at doses of 0, 2 or 8 mg/day, over 8 weeks in a double blind study and the effects on oxidative stress and inflammation were assessed [95]. The participants in this study did not have elevated oxidative stress and inflammation. However, those that took 2 mg/day had lower levels of C reactive protein by the eighth week. In addition, there was decreased DNA damage at four weeks of astaxanthin ingestion, assessed by measuring plasma 8-hydroxy-2′-deoxyguanosine. Orally administered astaxanthin reduced levels of oxidative stress biomarkers (malondialdehyde and isoprostanes) in healthy smokers [98]. Current evidence suggests astaxanthin is safe, orally bioavailable and suitable for further human studies.

#### Lipids and Metabolic Factors

Patients with moderate hypertriglyceridemia were treated with astaxanthin in a randomised controlled trial over 12 weeks [96]. There was a significant decrease in serum triglyceride levels and increase in serum HDL cholesterol levels in those treaded with 12 and 18 mg/day of astaxanthin. Serum adioponectin increased in those treated with 12 and 18 mg/day of orally administered astaxanthin. The changes in adiponectin correlated with those seen in HDL cholesterol.

In 23 obese and overweight healthy individuals both 5 and 20 mg/day of astaxanthin reduced biomarkers of oxidative stress including malondealdehyde, isoprostanes, superoxide dismutase, and total antioxidant capacity [97]. In another study in 27 obese and overweight patients, astaxanthin improved low-density lipoprotein cholesterol, apolipoprotein B and biomarkers of oxidative stress [99].

Seventeen volunteers at risk of developing the metabolic syndrome received astaxanthin 8 mg twice daily in an open labelled uncontrolled clinical study [94]. There was a significant increase in adiponectin levels and significant decreases in HbA1c and TNF-alpha in astaxanthin treated participants.

Thus, astaxanthin shows promise at improving a range of metabolic factors that may have benefits in cardiovascular risk reduction.

## 8. Ongoing Clinical Trial with Astaxanthin

The effects of 8 mg/day of orally administered astaxanthin derived from *haematoccocus pluvialis* are being investigated in a pilot randomised double blind placebo-controlled trial (Xanthin trial) [100]. In particular this trial is assessing the effects of astaxanthin on measures of oxidative stress, inflammation and vascular function in kidney transplant patients that are known to have increased cardiovascular disease risk [100].

The patients will have assessments of surrogate markers of cardiovascular disease that include arterial stiffness measured using aortic pulse wave velocity, augmentation index, brachial forearm reactivity and carotid artery intima-media thickness. The results from this pilot study may lead to the design of a larger trial in high-risk patients that will assess cardiovascular outcomes such as acute myocardial infarction and death.

## 9. Conclusions

Astaxanthin and derivatives have been documented to have cardiovascular protective effects after prior administration both orally and intravenously and across several animal species. It also reduces oxidative stress and inflammation, known contributors to the pathophysiology of atherosclerotic cardiovascular disease. However, currently it is not known whether astaxanthin has any primary or secondary therapeutic benefit in human atherosclerotic cardiovascular disease. Astaxanthin, being polar has theoretical benefits through strategic cell membrane placement and protection against oxidative stress by potently quenching singlet oxygen. It is possible through these important differences that astaxanthin may prove more beneficial at attenuating cardiovascular disease than previously investigated carotenoids such as β-carotene and other antioxidants such as vitamin E and vitamin C [24,25]. Well-designed clinical trials in patients at high cardiovascular risk and proven oxidative stress are important to establish the role of antioxidant therapy in atherosclerotic cardiovascular disease. There have been no significant adverse events reported in clinical trials in humans where astaxanthin has been administered. Orally administered astaxanthin has satisfactory bioavailability and efficacy at reducing oxidative stress and inflammation. This all sets the foundation for clinical studies in patients at high risk of atherosclerotic cardiovascular disease. 
